# Identification of the Key Fields and Their Key Technical Points of Oncology by Patent Analysis

**DOI:** 10.1371/journal.pone.0143573

**Published:** 2015-11-24

**Authors:** Ting Zhang, Juan Chen, Xiaofeng Jia

**Affiliations:** Institute of Medical Information & Library, Chinese Academy of Medical Sciences and Peking Union Medical College, Beijing, People’s Republic of China; Chinese Research Academy of Environmental Sciences, CHINA

## Abstract

**Background:**

This paper aims to identify the key fields and their key technical points of oncology by patent analysis.

**Methodology/Principal Findings:**

Patents of oncology applied from 2006 to 2012 were searched in the Thomson Innovation database. The key fields and their key technical points were determined by analyzing the Derwent Classification (DC) and the International Patent Classification (IPC), respectively. Patent applications in the top ten DC occupied 80% of all the patent applications of oncology, which were the ten fields of oncology to be analyzed. The number of patent applications in these ten fields of oncology was standardized based on patent applications of oncology from 2006 to 2012. For each field, standardization was conducted separately for each of the seven years (2006–2012) and the mean of the seven standardized values was calculated to reflect the relative amount of patent applications in that field; meanwhile, regression analysis using time (year) and the standardized values of patent applications in seven years (2006–2012) was conducted so as to evaluate the trend of patent applications in each field. Two-dimensional quadrant analysis, together with the professional knowledge of oncology, was taken into consideration in determining the key fields of oncology. The fields located in the quadrant with high relative amount or increasing trend of patent applications are identified as key ones. By using the same method, the key technical points in each key field were identified. Altogether 116,820 patents of oncology applied from 2006 to 2012 were retrieved, and four key fields with twenty-nine key technical points were identified, including “natural products and polymers” with nine key technical points, “fermentation industry” with twelve ones, “electrical medical equipment” with four ones, and “diagnosis, surgery” with four ones.

**Conclusions/Significance:**

The results of this study could provide guidance on the development direction of oncology, and also help researchers broaden innovative ideas and discover new technological opportunities.

## Introduction

Cancer is reported as the second major cause of deaths following cardiovascular disease and has become one of the leading threat worldwide [1,2]. With the development of sciences, the goal of curing cancer or prolonging the lives of cancer patients is achievable, but it is unable to curb the spread of crisis effectively just relying on the therapy. Therefore, it is very necessary to grasp the development direction of oncology, and discover new technological opportunities. The field of oncology develops rapidly and studies involving oncology is closely related to other subjects, including epidemiology, etiology and pathogenesis, drug discovery, diagnosis and treatment, surgery and so on [3–9]. In addition, studies related to oncology also involves scientometrics, such as publication quality [10], collaboration patterns [11], comparison of citation and usage indicators [12], and co-authorship network [13]. However, most of the scientometrics researches concerning oncology are mainly about external features, and there were few ones with regard to analyzing the inner characteristics. To our knowledge, there has been no identification and analysis of key fields and key technical points in oncology by patent analysis.

Patents are one of the results and performance in the form of technological innovation, and can reflect the level of technological innovation. Patent analysis is a primary informatics method used for analyzing key technology [14–16]. This study discusses the technology distribution and development trend of oncology based on patent analysis. The purpose was to identify the key fields of oncology and their key technical points by quantitative analysis including patentometrics, regression analysis and two-dimensional quadrant analysis, combined with qualitative analysis by oncology experts. The results can provide an objective statistical reference that government can use to optimize allocation of resources from the point of macro view, and also help researchers broaden innovative ideas and discover new technological opportunities.

## Materials and Methods

### Ethics Statement

This study is about the bibliometrics of oncology, and the subject investigated is patents of oncology. It does not involve human subject research or animal research, and no patient records/information and clinical records are included. Therefore, no ethics issue is involved in this research.

### Materials

Data were collected from the Thomson Innovation (TI) Database of Thomson Reuters using search strategy based on subject terms. Subject terms related to oncology were “neoplasm* OR tumor* OR cancer* OR neoplasia”. In this study, patents related to oncology applied from 2006 to 2012 were searched and collected from TI database, and then 116,820 patents were obtained. Patent data were imported into Thomson Data Analyzer 3.0 (TDA, Thomson Reuters Co., New York, NY, USA) for authority control, and input into SPSS 19.0 (SPSS Inc., Chicago, IL, USA) for regression analysis.

### Methods

Patent analysis is a primary informatics method used for analyzing key technology. The general status and development state of technology in a certain field can be revealed by analyzing the patents. Therefore, analyzing patents of oncology can uncover the development state and direction of advanced technologies, as well as unearth the potential economic value of a certain technology of oncology. Derwent Classification (DC) classifies patent documents with a simple classification system that is applicable to all kinds of technologies, which can help search technologies in a certain field efficiently and accurately. The patent classification system of International Patent Classification (IPC) can reflect the focus and research hotspots of a certain technology in some degree and has been widely used in the classification and retrieval of patent documents all over the world. In this study, DC and IPC were analyzed to identify the key fields and their key technical points respectively based on two-dimensional quadrant analysis.

Patents in the field of oncology applied from 2006 to 2012 were collected from TI database, and 116,820 patents were retrieved, including 287 DCs which represented the 287 fields. The numbers of patent applications in the top ten DC occupied 80% of the total numbers of patent applications in the field of oncology, which were chosen to identify the key fields. The number of patent applications in the ten fields of oncology was standardized based on the total numbers of oncology. For each field, standardization was conducted separately for seven years (2006–2012) and the mean of the seven standardized values (M, %) was calculated to reflect the relative amount of patent applications in that field; meanwhile, regression analysis using time (year) and the standardized amount (M, %) of patent applications in seven years (2006–2012) was conducted so as to evaluate the trend of patent applications in each field. Two-dimensional quadrant analysis was carried out based on the relative amount and the trend of patent applications.

In the two-dimensional quadrant analysis, the mean standardized value (M, %) was defined as the horizontal axis (X axis), and the regression coefficient *b* as the longitudinal axis (Y axis). M_*max*_ and M_*min*_ were the maximum and minimum of the mean standard values for the ten fields of oncology or the maximum and minimum of the mean standard values for the twenty technical points in each key field, respectively. For the X-axis, (M_*max*_ - M_*min*_)/2 was set as the cut-off point. The Y-axis indicated the coefficient *b* in the regression analysis, whose *P* values greater than 0.05 were set as 0 in the figure. Thus, [(M_*max*_ - M_*min*_)/2, 0] was set as the origin of the quadrant. The two-dimensional quadrant analysis showed that those fields or technical points located in I quadrant had a significant growth trend (*b*>0, *P*<0.05), those located in II quadrant both had a significant growth trend (*b*>0, *P*<0.05) and high mean standardized value [M>(M_*max*_ - M_*min*_)/2], and those on the coordinate axes of II–IV quadrant or located in IV quadrant had high mean standardized value [M>(M_*max*_ - M_*min*_)/2]. The fields or technical points located in above area were identified as key ones.

## Results

### The identification of key fields of oncology

Patents of oncology applied from 2006 to 2012 were collected from TI database, and were classified into 287 fields based on DC. The numbers of patent applications in the top ten DC occupied 80% of the total numbers of patent applications of oncology (Table 1), namely “natural products and polymers” (B04), “fermentation industry” (D16), “fused ring heterocyclics” (B02), “scientific instrumentation” (S03), “other organics” (B05), “medical, dental, veterinary, cosmetic” (A96), “general” (B07), “other heterocyclics” (B03), “electrical medical equipment” (S05), and “diagnosis, surgery” (P31), which were chosen as the analysis object of key fields identification.

**Table 1:** The patent applications in the top ten DC of oncology from 2006 to 2012.

No	DC	Meanings	Total	2006^a^	2007	2008	2009	2010	2011	2012
1	B04	Natural products and polymers	58897	7276 (50.18)	8175 (46.65)	10227 (50.49)	9089 (51.75)	9295 (53.30)	7574 (51.67)	7261 (50.63)
2	D16	Fermentation industry	46619	5559 (38.34)	6158 (35.14)	8184 (40.40)	7310 (41.62)	7680 (44.04)	5994 (40.90)	5734 (39.98)
3	B02	Fused ring heterocyclics	22374	2817 (19.43)	3922 (22.38)	4133 (20.40)	3569 (20.32)	3176 (18.21)	2402 (16.39)	2355 (16.42)
4	S03	Scientific instrumentation	16758	2495 (17.21)	2264 (12.92)	2902 (14.33)	2408 (13.71)	2529 (14.50)	2147 (14.65)	2013 (14.04)
5	B05	Other organics	16195	2041 (14.08)	2631 (15.01)	2923 (14.43)	2526 (14.38)	2265 (12.99)	1882 (12.84)	1927 (13.44)
6	A96	Medical, dental, veterinary, cosmetic	14036	1764 (12.17)	2296 (13.10)	2223 (10.97)	2111 (12.02)	2096 (12.02)	1785 (12.18)	1761 (12.28)
7	B07	General	13109	1495 (10.31)	2059 (11.75)	2265 (11.18)	2022 (11.51)	1880 (10.78)	1621 (11.06)	1767 (12.32)
8	B03	Other heterocyclics	12667	1730 (11.93)	2077 (11.85)	2507 (12.38)	1912 (10.89)	1849 (10.60)	1318 (8.99)	1274 (8.88)
9	S05	Electrical medical equipment	11876	1469 (10.13)	1651 (9.42)	1927 (9.51)	1812 (10.32)	1811 (10.39)	1645 (11.22)	1561 (10.88)
10	P31	Diagnosis, surgery	7956	955 (6.59)	1102 (6.29)	1224 (6.04)	1172 (6.67)	1224 (7.02)	1149 (7.84)	1130 (7.88)

Note:

^a^ The actual and standardized (%) number of patent applications in the ten fields of oncology applied in 2006. The standardized value = the yearly number of patent applications in each field divided by that in the whole field of oncology.

Two-dimensional quadrant analysis was used to identify the key fields of oncology. The annual number of patent applications in each of the top ten DC was divided by the total number of patent applications of oncology for standardization, and then the mean standardized value (M, %) of seven years (2006–2012) for each field was calculated (Table 2). Regression analysis was applied to discuss the trend of patents applications with time.

**Table 2:** The top ten DCs of oncology.

No	DC	Meanings	Mean standardized value (M)	*Coefficient b*	*P* value
1	**B04**	**Natural products and polymers**	**50.67**	*0*.*51*	0.22
2	**D16**	**Fermentation industry**	**40.06**	*0*.*72*	0.19
3	B02	Fused ring heterocyclics	19.08	-0.83	0.03
4	S03	Scientific instrumentation	14.48	*-0*.*21*	0.46
5	B05	Other organics	13.88	*-0*.*28*	0.06
6	A96	Medical, dental, veterinary, cosmetic	12.11	*-0*.*02*	0.90
7	B07	General	11.27	*0*.*15*	0.26
8	B03	Other heterocyclics	10.79	-0.59	0.00
9	**S05**	**Electrical medical equipment**	10.27	**0.24**	**0.04**
10	**P31**	**Diagnosis, surgery**	6.90	**0.28**	**0.01**

Note: *Coefficient b* and *P* value were obtained from the regression analysis with “the standardized number of patent applications for each year” as the dependent variable and “year” as the independent variable. Bold values refer to the values of the fields with a mean standardized value above (M_*max*_ - M_*min*_)/2 or with a significant growing trend (*P*<0.05 and *b*>0). Italic values refer to the fields whose *P* values are above 0.05, without statistical significance and their *coefficient b* values are defined 0 in the two-dimensional quadrant analysis.

**Fig 1:**
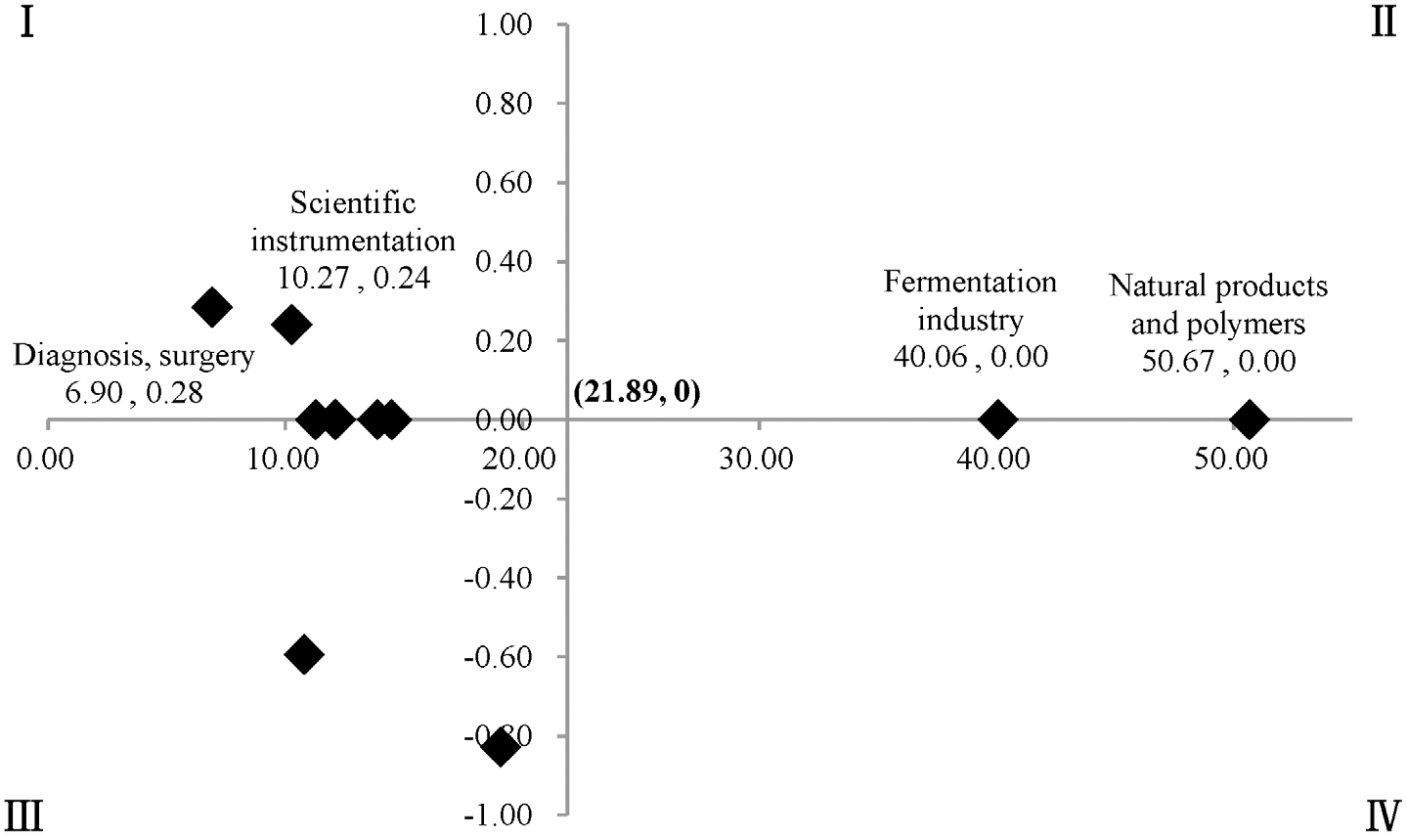
The key fields of oncology. The X-axis indicated (M_*max*_ - M_*min*_)/2, namely M = 21.89, as cut-off point. The Y-axis indicated the *coefficient b* in the regression analysis, whose *P* values of difference-test greater than 0.05, are set as 0 in the figure, and (21.89, 0) was set as the origin of the quadrant.

In the two-dimensional quadrant analysis, the mean standardized value (M, %) was defined as the horizontal axis (X axis), and M_*max*_ and M_*min*_ were the maximum and minimum of the mean standard values for the ten fields of oncology, namely the M of “natural products and polymers” (B04) and “diagnosis, surgery” (P31), respectively. For the X-axis, (M_*max*_ - M_*min*_)/2, namely M = (50.67–6.90)/2 = 21.89, was set as the cut-off point, and then (21.89, 0) was set as the origin of the quadrant (Fig 1).

The two-dimensional quadrant analysis showed that “natural products and polymers” (B04) and “fermentation industry” (D16) located on the coordinate axes of II–IV quadrant, with a high mean standardized value (M = 50.57, 40.06, respectively, above 21.89); “electronic medical equipment” (S05) and “diagnosis, surgery” (P31) located in I quadrant, with a significant growth trend (*b* = 0.24, 0.28, respectively, *P*<0.05). Consequently, there were four key fields of oncology including “natural products and polymers” (B04), “fermentation industry” (D16), “electrical medical equipment” (S05), and “diagnosis, surgery” (P31).

### The identification of key technical points in each key field of oncology

The patents in each key field of oncology were analyzed based on IPC. IPCs with top twenty numbers of patent applications in each key field of oncology refer to the top twenty technical points. The key technical points were identified by the same methods as identifying the four key fields of oncology. The number of patent applications with the top twenty IPCs in each key field was standardized based on the number of patent applications in the corresponding key field for each year, respectively. Then the mean standardized value (M, %) of seven years (2006–2012) was calculated, and the standardized values were analyzed by regression analysis. The two-dimensional quadrant analysis showed that those technical points in I, II or IV quadrant or on the coordinate axes of II–IV quadrant were the key ones.

#### The identification of key technical points in the field of “natural products and polymers” of oncology

The annual number of patent applications with the top twenty IPCs was divided by that in the field of “natural products and polymers” of oncology for standardization, and then the mean standardized value (M, %) of seven years (2006–2012) was calculated for each field (Table 3). Regression analysis was applied to discuss the trend of patents applications with time.

**Table 3:** The top twenty IPCs in the field of “natural products and polymers” of oncology.

No	IPC	Meanings	Numbers	Mean standardized value (M)	*Coefficient b*	*P* value
1	**A61P-0035**	Antineoplastic agents	15709	**26.68**	**2.16**	**0.00**
2	**G01N-0033**	Investigating or analysing materials by specific methods not covered by the preceding groups	11203	**19.02**	*0*.*21*	0.17
3	**C12Q-0001**	Measuring or testing processes involving enzymes or micro-organisms; Compositions therefor; Processes of preparing such compositions	10631	**18.09**	*0*.*14*	0.34
4	**C12N-0015**	Mutation or genetic engineering; DNA or RNA concerning genetic engineering, vectors, e.g. plasmids, or their isolation, preparation or purification; Use of hosts therefor	9757	**16.55**	*0*.*01*	0.97
5	**A61K-0038**	Medicinal preparations containing peptides	8998	**15.16**	-0.67	0.03
6	**A61K-0039**	Medicinal preparations containing antigens or antibodies	8807	**14.89**	-0.40	0.03
7	**A61K-0031**	Medicinal preparations containing organic active ingredients	8764	**14.81**	*0*.*17*	0.57
8	C07K-0016	Immunoglobulins, e.g. monoclonal or polyclonal antibodies	5784	9.78	*-0*.*17*	0.17
9	A61K-0036	Medicinal preparations of undetermined constitution containing material from algae, lichens, fungi or plants, or derivatives thereof, e.g. traditional herbal medicines	5659	9.73	*0*.*44*	0.08
10	C07K-0014	Peptides having more than 20 amino acids; Gastrins; Somatostatins; Melanotropins; Derivatives thereof	5549	9.40	-1.00	0.00
11	C12N-0005	Undifferentiated human, animal or plant cells, e.g. cell lines; Tissues; Cultivation or maintenance thereof; Culture media therefor	5433	9.22	*0*.*09*	0.64
12	A61K-0048	Medicinal preparations containing genetic material which is inserted into cells of the living body to treat genetic diseases; Gene therapy	3909	6.69	*-0*.*31*	0.06
13	A61K-0035	Medicinal preparations containing material or reaction products thereof with undetermined constitution	3639	6.21	*0*.*11*	0.35
14	**A61K-0009**	Medicinal preparations characterised by special physical form	3190	5.45	**0.22**	**0.02**
15	**A61K-0047**	Medicinal preparations characterised by the non-active ingredients used, e.g. carriers, inert additives	3094	5.28	**0.34**	**0.01**
16	A61P-0037	Drugs for immunological or allergic disorders	3026	5.02	*-0*.*03*	0.91
17	A61P-0031	Antiinfectives, i.e. antibiotics, antiseptics, chemotherapeutics	2526	4.17	*0*.*05*	0.87
18	A61P-0009	Drugs for disorders of the cardiovascular system	2041	3.33	*-0*.*03*	0.91
19	C07H-0021	Compounds containing two or more mononucleotide units having separate phosphate or polyphosphate groups linked by saccharide radicals of nucleoside groups, e.g. nucleic acids	1944	3.28	-0.44	0.00
20	A61P-0025	Drugs for disorders of the nervous system	1941	3.18	*0*.*05*	0.87

Note: *Coefficient b* and *P* value were obtained from the regression analysis with “the standardized number of patent applications for each year” as the dependent variable and “year” as the independent variable. Bold values refer to the values of subfields with a mean standardized value above (M_*max*_ - M_*min*_)/2 or with a significant growing trend (*P*<0.05 and *b*>0). Italic values refer to the fields whose *P* values are above 0.05, without statistical significance and their *coefficient b* values are defined 0 in the two-dimensional quadrant analysis.

**Fig 2:**
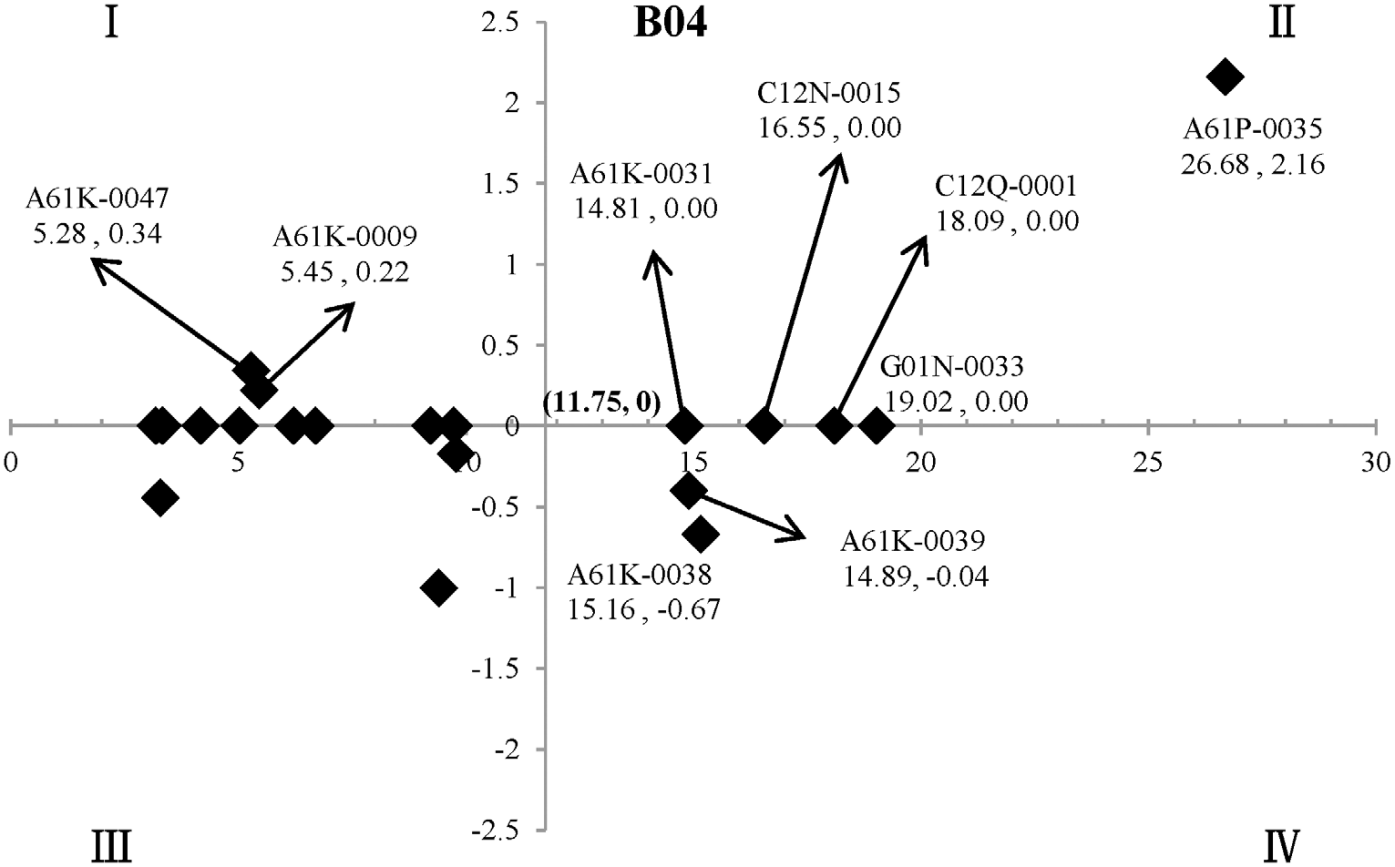
The key technical points in the field of “natural products and polymers” of oncology. The X-axis indicated (M_*max*_ - M_*min*_)/2, namely M = 11.75, as cut-off point. The Y-axis indicated the *coefficient b* in the regression analysis, whose *P* values of difference-test greater than 0.05, are set as 0 in the figure, and (11.75, 0) was set as the origin of the quadrant.

In the two-dimensional quadrant analysis, M_*max*_ and M_*min*_ were the maximum and minimum of the mean standard values (M, %) for the twenty technical points in the field of “natural products and polymers” of oncology, namely the M of A61P-0035 (M = 26.68) and A61P-0025 (M = 3.18), respectively. For the X-axis, (M_*max*_ - M_*min*_)/2, namely M = (26.68–3.18)/2 = 11.75, was set as the cut-off point, and then (11.75, 0) was set as the origin of the quadrant (Fig 2).

The two-dimensional quadrant analysis showed that there were nine key technical points in the field of “natural products and polymers” of oncology, namely A61P-0035 (Antineoplastic agents), G01N-0033 (Investigating or analysing materials by specific methods not covered by the preceding groups), C12Q-0001 (Measuring or testing processes involving enzymes or micro-organisms; Compositions therefor; Processes of preparing such compositions), C12N-0015 (Mutation or genetic engineering; DNA or RNA concerning genetic engineering, vectors, e.g. plasmids, or their isolation, preparation or purification; Use of hosts therefor), A61K-0038 (Medicinal preparations containing peptides), A61K-0039 (Medicinal preparations containing antigens or antibodies), A61K-0031 (Medicinal preparations containing organic active ingredients), A61K-0009 (Medicinal preparations characterised by special physical form) and A61K-0047 (Medicinal preparations characterised by the non-active ingredients used, e.g. carriers, inert additives).

#### The identification of key technical points in the field of “fermentation industry” of oncology

The annual number of patent applications with the top twenty IPCs was divided by that in the field of “fermentation industry” of oncology for standardization, and then the mean standardized value (M, %) of seven years (2006–2012) was calculated for each field (Table 4). Regression analysis was applied to discuss the trend of patents applications with time.

**Table 4:** The top twenty IPCs in the field of “fermentation industry” of oncology.

No	IPC	Meanings	Numbers	Mean standardized value (M)	*Coefficient b*	*P* value
1	**A61P-0035**	Antineoplastic agents	10954	**23.31**	**2.19**	**0.01**
2	**C12Q-0001**	Measuring or testing processes involving enzymes or micro-organisms; Compositions therefor; Processes of preparing such compositions	10654	**22.96**	*0*.*05*	0.80
3	**G01N-0033**	Investigating or analysing materials by specific methods not covered by the preceding groups	10476	**22.53**	*0*.*13*	0.43
4	**C12N-0015**	Mutation or genetic engineering; DNA or RNA concerning genetic engineering, vectors, e.g. plasmids, or their isolation, preparation or purification; Use of hosts therefor	9906	**21.27**	*-0*.*02*	0.94
5	**A61K-0039**	Medicinal preparations containing antigens or antibodies	8656	**18.52**	-0.48	0.02
6	**A61K-0038**	Medicinal preparations containing peptides	7513	**15.99**	*-0*.*39*	0.25
7	**A61K-0031**	Medicinal preparations containing organic active ingredients	6567	**13.92**	*0*.*67*	0.14
8	**C07K-0016**	Immunoglobulins, e.g. monoclonal or polyclonal antibodies	5775	**12.36**	-0.28	0.04
9	**C12N-0005**	Undifferentiated human, animal or plant cells, e.g. cell lines; Tissues; Cultivation or maintenance thereof; Culture media therefor	5476	**11.76**	*0*.*08*	0.73
10	**C07K-0014**	Peptides having more than 20 amino acids; Gastrins; Somatostatins; Melanotropins; Derivatives thereof	5187	**11.15**	-1.14	0.00
11	A61K-0048	Medicinal preparations containing genetic material which is inserted into cells of the living body to treat genetic diseases; Gene therapy	3791	8.23	*-0*.*33*	0.11
12	A61P-0037	Drugs for immunological or allergic disorders	2315	4.82	*0*.*02*	0.96
13	**A61K-0047**	Medicinal preparations characterised by the non-active ingredients used, e.g. carriers, inert additives	2259	4.84	**0.48**	**0.00**
14	A61P-0031	Antiinfectives, i.e. antibiotics, antiseptics, chemotherapeutics	1934	4.00	*0*.*12*	0.74
15	C07H-0021	Compounds containing two or more mononucleotide units having separate phosphate or polyphosphate groups linked by saccharide radicals of nucleoside groups, e.g. nucleic acids	1852	3.98	-0.57	0.01
16	A61K-0035	Medicinal preparations containing material or reaction products thereof with undetermined constitution	1843	3.97	*0*.*08*	0.44
17	C12P-0021	Preparation of peptides or proteins	1739	3.76	-0.55	0.00
18	C12N-0001	Micro-organisms, e.g. protozoa; Compositions thereof; Processes of propagating, maintaining or preserving micro-organisms or compositions thereof; Processes of preparing or isolating a composition containing a micro-organism; Culture media therefor	1613	3.46	*-0*.*03*	0.69
19	**A61K-0009**	Medicinal preparations characterised by special physical form	1589	3.38	**0.47**	**0.00**
20	A61P-0025	Drugs for disorders of the nervous system	1417	2.89	*0*.*09*	0.79

Note: *Coefficient b* and *P* value were obtained from the regression analysis with “the standardized number of patent applications for each year” as the dependent variable and “year” as the independent variable. Bold values refer to the values of subfields with a mean standardized value above (M_*max*_ - M_*min*_)/2 or with a significant growing trend (*P*<0.05 and *b*>0). Italic values refer to the fields whose *P* values are above 0.05, without statistical significance and their *coefficient b* values are defined 0 in the two-dimensional quadrant analysis.

**Fig 3:**
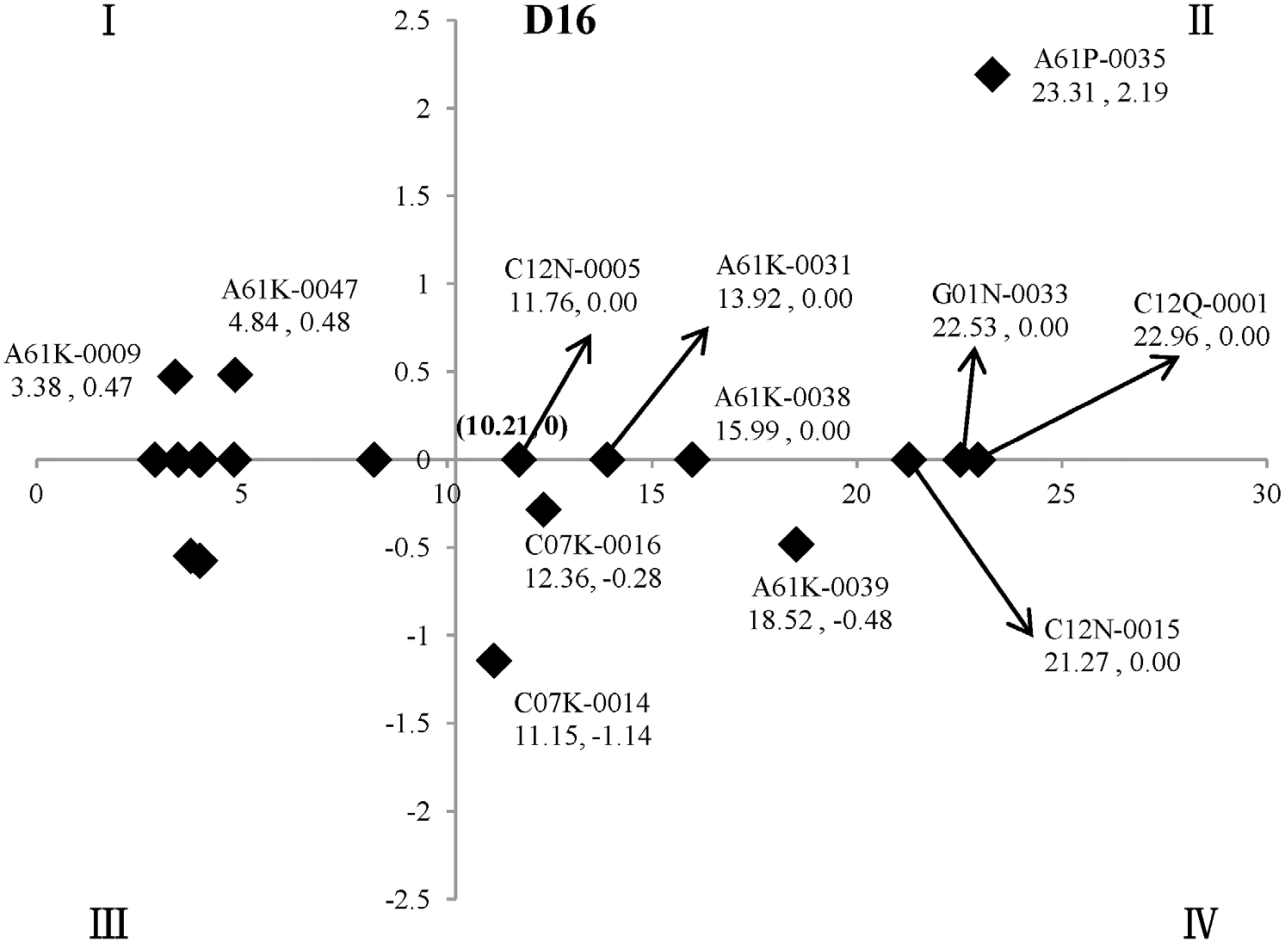
The key technical points in the field of “fermentation industry” of oncology. The X-axis indicated (M_*max*_ - M_*min*_) /2, namely M = 10.21, as cut-off point. The Y-axis indicated the *coefficient b* in the regression analysis, whose *P* values of difference-test greater than 0.05, are set as 0 in the figure, and (10.21, 0) was set as the origin of the quadrant.

In the two-dimensional quadrant analysis, M_*max*_ and M_*min*_ were the maximum and minimum of the mean standard values (M, %) for the twenty technical points in the field of “fermentation industry” of oncology, namely the M of A61P-0035 (M = 23.31) and A61P-0025 (M = 2.89), respectively. For the X-axis, (M_*max*_ - M_*min*_)/2, namely M = (23.31–2.89)/2 = 10.21, was set as the cut-off point, and then (10.21, 0) was set as the origin of the quadrant (Fig 3).

The two-dimensional quadrant analysis showed that there were twelve key technical points in the field of “fermentation industry” of oncology, namely A61P-0035, C12Q-0001, G01N-0033, C12N-0015, A61K-0039, A61K-0038, A61K-0031, C07K-0016 (Immunoglobulins, e.g. monoclonal or polyclonal antibodies), C12N-0005 (Undifferentiated human, animal or plant cells, e.g. cell lines; Tissues; Cultivation or maintenance thereof; Culture media therefor), C07K-0014 (Peptides having more than 20 amino acids; Gastrins; Somatostatins; Melanotropins; Derivatives thereof), A61K-0047 and A61K-0009.

#### The identification of key technical points in the field of “electrical medical equipment” of oncology

The annual number of patent applications with the top twenty IPCs was divided by that in the field of “electrical medical equipment” of oncology for standardization, and then the mean standardized value (M, %) of seven years (2006–2012) was calculated for each field (Table 5). Regression analysis was applied to discuss the trend of patents applications with time.

**Table 5:** The top twenty IPCs in the field of “electrical medical equipment” of oncology.

No	IPC	Meanings	Numbers	Mean standardized value (M)	*Coefficient b*	*P* value
1	**A61N-0005**	Radiation therapy	2180	**18.33**	**1.02**	**0.00**
2	**A61B-0005**	Measuring for diagnostic purposes; Identification of persons	1715	**14.45**	*-0*.*15*	0.19
3	**A61B-0006**	Apparatus for radiation diagnosis, e.g. combined with radiation therapy equipment	1321	**11.15**	*0*.*46*	0.18
4	**A61B-0018**	Surgical instruments, devices or methods for transferring non-mechanical forms of energy to or from the body	1117	**9.46**	*-0*.*05*	0.85
5	A61B-0008	Diagnosis using ultrasonic, sonic or infrasonic waves	912	7.70	*0*.*11*	0.37
6	A61K-0031	Medicinal preparations containing organic active ingredients	699	5.80	*-0*.*31*	0.36
7	A61P-0035	Antineoplastic agents	625	5.21	*0*.*21*	0.27
8	A61N-0001	Electrotherapy; Circuits therefor	564	4.78	-0.43	0.01
9	A61B-0001	Instruments for performing medical examinations of the interior of cavities or tubes of the body by visual or photographical inspection, e.g. endoscopes; Illuminating arrangements therefor	468	3.93	*0*.*16*	0.18
10	G01N-0033	Investigating or analysing materials by specific methods not covered by the preceding groups	462	3.86	*-0*.*06*	0.81
11	A61B-0017	Surgical instruments, devices or methods, e.g. tourniquets	439	3.71	*0*.*13*	0.30
12	A61K-0049	Preparations for testing in vivo	436	3.69	-0.33	0.01
13	A61B-0019	Instruments, implements or accessories for surgery or diagnosis not covered by any of the groups—see cross reference IPC A61B-001/00 to—see cross reference IPC A61B-018/00, e.g. for stereotaxis, sterile operation, luxation treatment, wound edge protectors	417	3.49	*-0*.*06*	0.58
14	G06F-0019	Digital computing or data processing equipment or methods, specially adapted for specific applications	401	3.41	*0*.*14*	0.32
15	G06K-0009	Methods or arrangements for reading or recognising printed or written characters or for recognising patterns, e.g. fingerprints	363	3.05	-0.23	0.04
16	A61N-0007	Ultrasound therapy	326	2.74	*0*.*05*	0.70
17	G01N-0021	Investigating or analysing materials by the use of optical means, i.e. using infra-red, visible, or ultra-violet light	312	2.65	*0*.*03*	0.69
18	A61K-0051	Preparations containing radioactive substances for use in therapy or testing in vivo	283	2.38	-0.25	0.02
19	C12Q-0001	Measuring or testing processes involving enzymes or micro-organisms; Compositions therefor; Processes of preparing such compositions	282	2.39	*-0*.*07*	0.33
20	G06T-0007	Image analysis, e.g. from bit-mapped to non bit-mapped	276	2.31	*-0*.*05*	0.65

Note: *Coefficient b* and *P* value were obtained from the regression analysis with “the standardized number of patent applications for each year” as the dependent variable and “year” as the independent variable. Bold values refer to the values of subfields with a mean standardized value above (M_*max*_ - M_*min*_)/2 or with a significant growing trend (*P*<0.05 and *b*>0). Italic values refer to the fields whose *P* values are above 0.05, without statistical significance and their *coefficient b* values are defined 0 in the two-dimensional quadrant analysis.

**Fig 4:**
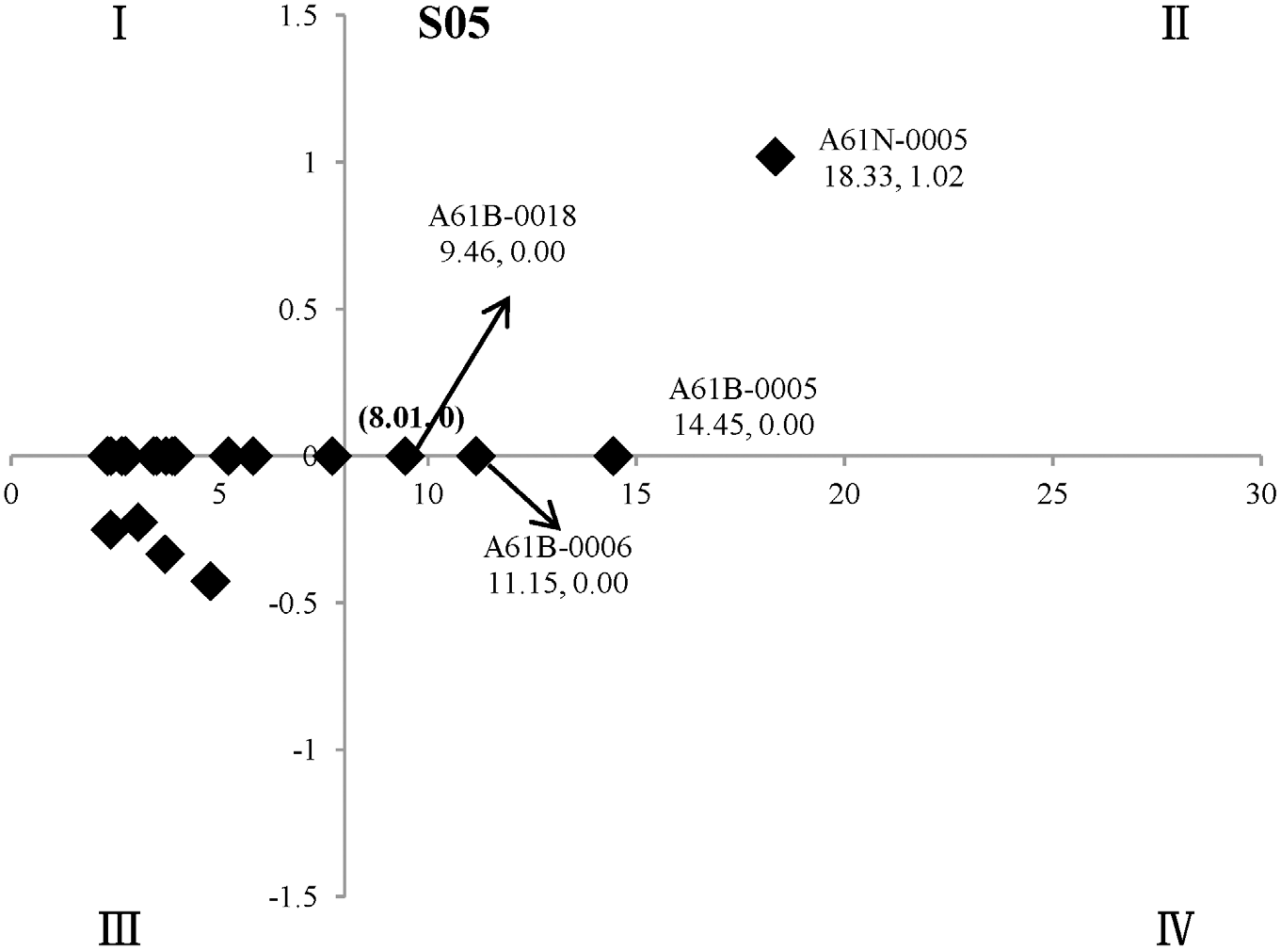
The key technical points in the field of “electrical medical equipment” of oncology. The X-axis indicated (M_*max*_ - M_*min*_) /2, namely M = 8.01, as cut-off point. The Y-axis indicated the *coefficient b* in the regression analysis, whose *P* values of difference-test greater than 0.05, are set as 0 in the figure, and (8.01, 0) was set as the origin of the quadrant.

In the two-dimensional quadrant analysis, M_*max*_ and M_*min*_ were the maximum and minimum of the mean standard values (M, %) for the twenty technical points in the field of “electrical medical equipment” of oncology, namely the M of A61N-0005 (M = 18.33) and G06T-0007 (M = 2.31), respectively. For the X-axis, (M_*max*_ - M_*min*_)/2, namely M = (18.33–2.31)/2 = 8.01, was set as the cut-off point, and then (8.01, 0) was set as the origin of the quadrant (Fig 4).

The two-dimensional quadrant analysis showed that there were four key technical points in the field of “electrical medical equipment” of oncology, namely A61N-0005 (Radiation therapy), A61B-0005 (Measuring for diagnostic purposes; Identification of persons), A61B-0006 (Apparatus for radiation diagnosis, e.g. combined with radiation therapy equipment) and A61B-0018 (Surgical instruments, devices or methods for transferring non-mechanical forms of energy to or from the body).

#### The identification of key technical points in the field of “diagnosis, surgery” of oncology

The annual number of patent applications with the top twenty IPCs was divided by that in the field of “diagnosis, surgery” of oncology for standardization, and then the mean standardized value (M, %) of seven years (2006–2012) was calculated for each field (Table 6). Regression analysis was applied to discuss the trend of patents applications with time.

**Table 6:** The top twenty IPCs in the field of “diagnosis, surgery” of oncology.

No	IPC	Meanings	Numbers	Mean standardized value (M)	*Coefficient b*	*P* value
1	**A61B-0005**	Measuring for diagnostic purposes; Identification of persons	2145	**26.90**	*-0*.*49*	0.24
2	**A61B-0017**	Surgical instruments, devices or methods, e.g. tourniquets	1674	**20.98**	**1.17**	**0.00**
3	**A61B-0006**	Apparatus for radiation diagnosis, e.g. combined with radiation therapy equipment	1442	**18.11**	*0*.*52*	0.27
4	**A61B-0018**	Surgical instruments, devices or methods for transferring non-mechanical forms of energy to or from the body	1355	**17.10**	*-0*.*48*	0.24
5	A61B-0008	Diagnosis using ultrasonic, sonic or infrasonic waves	979	12.33	*-0*.*13*	0.37
6	A61B-0001	Instruments for performing medical examinations of the interior of cavities or tubes of the body by visual or photographical inspection, e.g. endoscopes; Illuminating arrangements therefor	690	8.64	*0*.*04*	0.84
7	A61B-0010	Other methods or instruments for diagnosis, e.g. instruments for taking a cell sample, for biopsy, for vaccination diagnosis; Sex determination; Ovulation-period determination; Throat striking implements	683	8.56	*0*.*24*	0.08
8	A61B-0019	Instruments, implements or accessories for surgery or diagnosis not covered by any of the groups—see cross reference IPC A61B-001/00 to—see cross reference IPC A61B-018/00, e.g. for stereotaxis, sterile operation, luxation treatment, wound edge protectors	580	7.26	*-0*.*10*	0.69
9	A61N-0005	Radiation therapy	522	6.59	*0*.*00*	1.00
10	G01N-0021	Investigating or analysing materials by the use of optical means, i.e. using infra-red, visible, or ultra-violet light	249	3.13	*0*.*02*	0.76
11	G01N-0033	Investigating or analysing materials by specific methods not covered by the preceding groups	204	2.53	*0*.*00*	0.99
12	A61F-0002	Filters implantable into blood vessels; Prostheses, i.e. artificial substitutes or replacements for parts of the body; Appliances for connecting them with the body	182	2.29	*-0*.*26*	0.06
13	A61N-0007	Ultrasound therapy	173	2.16	*0*.*04*	0.73
14	A61K-0049	Preparations for testing in vivo	142	1.78	*-0*.*12*	0.30
15	G06T-0007	Image analysis, e.g. from bit-mapped to non bit-mapped	140	1.76	*0*.*02*	0.74
16	A61M-0025	Catheters; Hollow probes	122	1.54	*0*.*12*	0.27
17	G01R-0033	Arrangements or instruments for measuring magnetic variables	121	1.52	*-0*.*08*	0.40
18	A61N-0001	Electrotherapy; Circuits therefor	118	1.49	*-0*.*03*	0.75
19	A61P-0035	Antineoplastic agents	116	1.45	*-0*.*02*	0.83
20	G01T-0001	Measuring X-radiation, gamma radiation, corpuscular radiation, or cosmic radiation	105	1.34	*0*.*07*	0.51

Note: *Coefficient b* and *P* value were obtained from the regression analysis with “the standardized number of patent applications for each year” as the dependent variable and “year” as the independent variable. Bold values refer to the values of subfields with a mean standardized value above (M_*max*_ - M_*min*_)/2 or with a significant growing trend (*P*<0.05 and *b*>0). Italic values refer to the fields whose *P* values are above 0.05, without statistical significance and their *coefficient b* values are defined 0 in the two-dimensional quadrant analysis.

**Fig 5:**
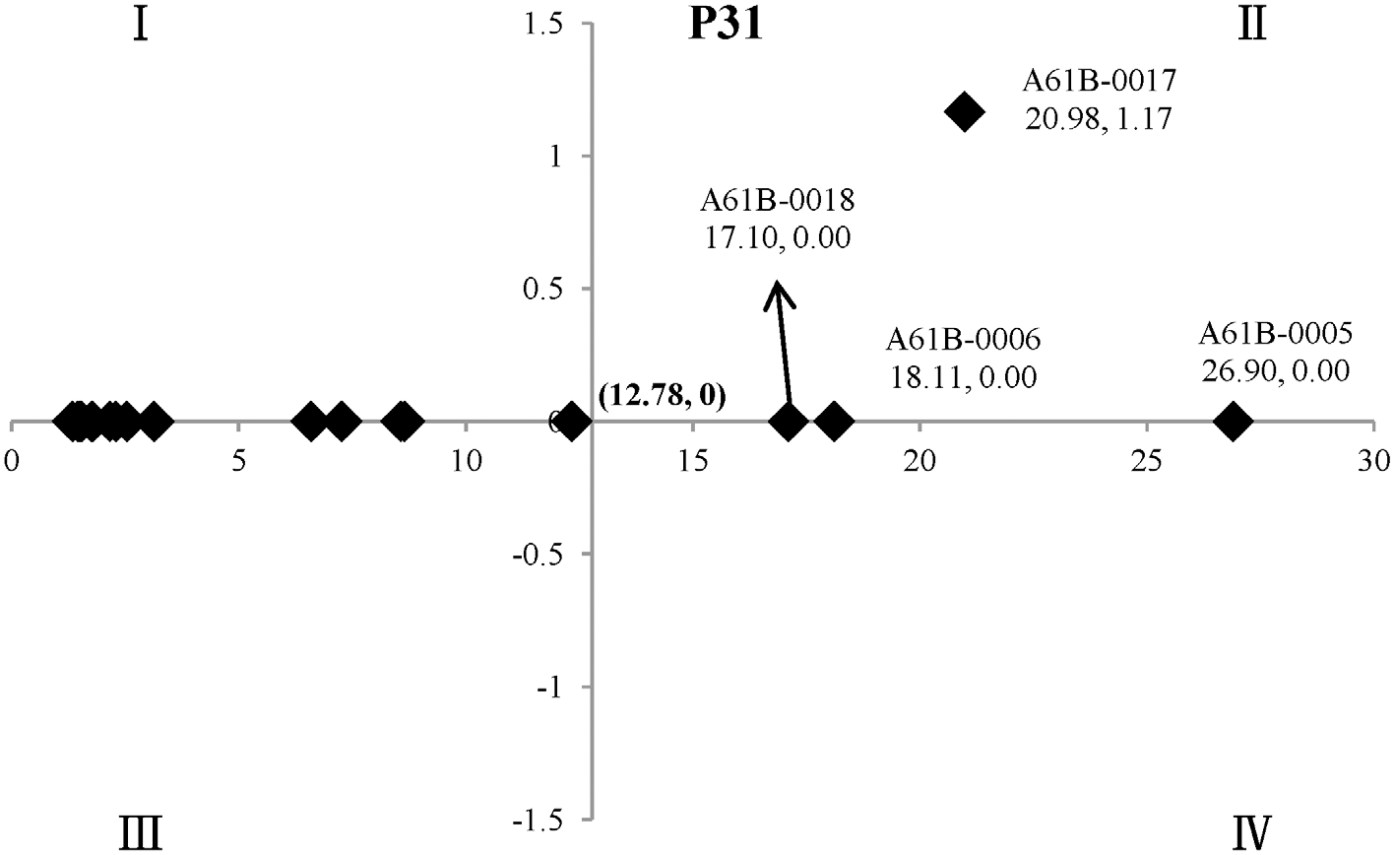
The key technical points in the field of “diagnosis, surgery” of oncology. The X-axis indicated (M_*max*_ - M_*min*_) /2, namely M = 12.78, as cut-off point. The Y-axis indicated the *coefficient b* in the regression analysis, whose *P* values of difference-test greater than 0.05, are set as 0 in the figure, and (12.78, 0) was set as the origin of the quadrant.

In the two-dimensional quadrant analysis, M_*max*_ and M_*min*_ were the maximum and minimum of the mean standard values (M, %) for the twenty technical points in the field of “diagnosis, surgery” of oncology, namely the M of A61B-0005 (M = 26.90) and G01T-0001 (M = 1.34), respectively. For the X-axis, (M_*max*_ - M_*min*_)/2, namely M = (26.90–1.34)/2 = 12.78, was set as the cut-off point, and then (12.78, 0) was set as the origin of the quadrant (Fig 5).

The two-dimensional quadrant analysis showed that there were four key technical points in the field of “diagnosis, surgery” of oncology, namely A61B-0005, A61B-0017 (Surgical instruments, devices or methods, e.g. tourniquets), A61B-0006 and A61B-0018.

## Discussion

### The analysis of key technical points in the fields of “natural products and polymers” and “fermentation industry” of oncology

Nine key technical points were the same in the fields of “natural products and polymers” and “fermentation industry” of oncology, namely A61P-0035, G01N-0033, C12Q-0001, C12N-0015, A61K-0038, A61K-0039, A61K-0031, A61K-0009 and A61K-0047, which indicated that most technical points in the two fields were interlinked, mainly related to activities (A61P-0035), preparations (A61K-0038, A61K-0039, A61K-0031, A61K-0009and A61K-0047), biological technology (C12Q-0001 and C12N-0015) and special methods (G01N-0033).

The reason why the key technical points are almost the same in these two fields is that microbial fermentation technology has been widely used in natural products research at present. In addition to extract and isolate chemical ingredients directly from natural products to obtain new drugs, it is necessary to carry on structural modification of chemical composition, and microbial synthesis is an important way. For example, biosynthesis occupies an important position in taxol research, which has also achieved gratifying results [17–20]. Studies on biosynthetic pathway of natural products is a very important aspect in biosynthetic research, which also involved discovering compounds with potential medical value by using modern cytology, genetics, physiology, and a variety of modern biotechnology. It is important to synthesize genes associated with natural active ingredients from natural products by biosynthesis or control expression of functional gene in the biosynthesis of natural products, which are further promoted by the achievements made by human genome research. It has made great progress to culture valuable products by using microbial, plant or animal cells and tissues or to obtain them by directly using enzyme to make structural modification of compounds, such as drug discovery through biological transformation [21–23].

A61P-0035, with the meaning of “antineoplastic agents” and related to the studies of pharmaceutical activity, not only ranks first, but also has a significant growing trend (*P*<0.05 and *b*>0), in both the key fields of “natural products and polymers” and “fermentation industry” of oncology, which indicates that A61P-0035 is one of the most important technical points. Finding antineoplastic active ingredients is an important area of discovering new drugs and antineoplastic agents has been one of the hot spots in new drug discovery all the time. Searching chemical ingredients with antineoplastic activity from natural products is an important way in drug discovery. With novel structure and various pharmacological activities, natural products are the research direction of pharmacy of concern all over the world, and many novel chemical compositions with antineoplastic activity were isolated from natural products [24–27]. Natural products cover a variety of species distributed from land to ocean, and medicinal plants, Chinese herbal medicine and marine organisms, especially marine organisms, are important sources for natural products. With marine organisms living in special environment, a large amount of substances with unique and novel structures as well as special biological activity are accumulated in the process of growing and metabolism [28,29]. Therefore, marine natural products are important resources for novel marine drugs, which will be effective for many kinds of diseases. Researches for marine drugs are currently in the ascendant, making this kind of drugs being more and more important. “Fermentation industry”, using microbial fermentation to discover new drugs, which enables enzyme to catalyze the specific site of specific substrate precisely, in consequence of higher selectivity bringing about no by-products, is an important direction of drug discovery [30,31]. Furthermore, the biosynthetic potential of endophytes in traditional Chinese anticancer herbs was investigated. Traditional Chinese medicine encompasses a rich empirical knowledge of using plants for the treatment of disease. In addition, the microorganisms associated with medicinal plants are also of interest as the producers of the compounds responsible for the observed plant bioactivity [32].

There are five key technical points about preparations (A61K-0038, A61K-0039, A61K-0031, A61K-0009 and A61K-0047) in the same nine ones in the two key fields. Medicine must be prepared to be suitable preparations for clinical use. The purpose of preparation is to ensure drugs safety, efficacy, stability and convenience. If the preparations are not appropriate, or the designation of prescription and process is unreasonable, the quality of products, even the efficacy and safety, will be affected. Therefore, the preparation research plays an important role in drug discovery [33,34]. It should be noted that A61K-0009and A61K-0047 are not about technical research on the preparations of active ingredients, but some external characteristics of preparations, such as appearance and shape. Preparation research is very broad, not only involving preparation technology itself, but also including designation of appearance, shape, route and other related research [35,36]. A61K-0038 and A61K-0039 are related to peptides and vaccines against cancer, respectively. Peptides with antineoplastic activity have become a new research focus of treating cancer [37,38]. Due to the characteristics of small molecular weight, more endogenous target, penetrating the tumor cells easily, improving immune response, inhibition of tumor angiogenesis, growth and metastasis and so on, peptides have shown promise in pre-clinical and clinical studies for cancer [39,40].

Tumor vaccines, also called active specific immunotherapy into tumors, are one of the hotspots in recent years [41,42], the principle of which is to eradicate or control tumor cells by means of activating the patients’ own immune system. Few tumor vaccines have been listed, most of which are in fundamental research or clinical trials. Although vaccine industry is just emerging, it has shown very strong momentum of development [43]. The advantage of tumor vaccines is that it could produce long-term immune memory and more durable antitumor effect. In addition to the same nine key technical points, there are another three ones in “fermentation industry”, namely C07K-0016, C12N-0005 and C07K-0014, which are related to tumor immunology, tumor cell culture, screening antineoplastic agents through genetic engineering, respectively.

### The analysis of key technical points in the fields of “electrical medical equipment” and “diagnosis, surgery” of oncology

Three same key technical points, including A61B-0005, A61B-0006 and A61B-0018, were in the fields of “electrical medical equipment” and “diagnosis, surgery” of oncology, the first two of which are related to diagnosis technology, and the last one is about surgical instruments, devices and methods. All of the three key technical points have large number of patent applications in the two key fields, which indicate that they are important research directions.

Except the above-mentioned three ones, there is another key technical point (A61N-0005, radiation therapy) in the field of “electrical medical equipment”. The technical point A61N-0005 not only ranks first, but also has a significant growing trend (*P*<0.05 and *b*>0), indicating that it has good prospects for development, and occupies an important position in cancer therapy. Radiation therapy, using radiation to kill tumor cells to control the growth and spread of cancer cells, is one of the primary means of treating malignant tumors. With continuing replacement of therapy equipments and emergence of new technologies, the effect of radiation has also been greatly improved [44–46]. With the improvement and development of new technologies in radiotherapy, a variety of fusion between radiotherapy techniques will drive future cancer radiotherapy toward real-time, high-precision-oriented direction.

A61B-0017, mostly related to surgical consumables, is another key technical point in the field of “diagnosis, surgery”, which ranks second and has a significant growing trend (*P*<0.05 and *b*>0) meanwhile. Surgery is still the preferred method of the current treatment of malignant tumors, which is the most promising development direction of oncology.

## Conclusion

By comprehensively applying quantitative and qualitative analysis, four key fields with twenty-nine key technical points of oncology were identified, including “natural products and polymers” with nine ones, “fermentation industry” with twelve ones, “electrical medical equipment” with four ones and “diagnosis, surgery” with four ones (Table 7). Nine key technical points were the same for the key fields of “natural products and polymers” and “fermentation industry” of oncology, and three ones were the same for the key fields of “electrical medical equipment” and “diagnosis, surgery” of oncology, indicating that many technical points were interlinked. The same key technical points in two fields need more concern.

**Table 7:** The four key fields and their key technical points of oncology.

Key Fields (DC)	Key Technical points (IPC)	Mean standardized value (M)	*Coefficient b*	*P* value
Natural products and polymers	A61P-0035	**26.68**	**2.16**	**0.00**
(B04)	G01N-0033	**19.02**	*0*.*21*	0.17
	C12Q-0001	**18.09**	*0*.*14*	0.34
	C12N-0015	**16.55**	*0*.*01*	0.97
	A61K-0038	**15.16**	-0.67	0.03
	A61K-0039	**14.89**	-0.40	0.03
	A61K-0031	**14.81**	*0*.*17*	0.57
	A61K-0009	5.45	**0.22**	**0.02**
	A61K-0047	5.28	**0.34**	**0.01**
Fermentation industry	A61P-0035	**23.31**	**2.19**	**0.01**
(D16)	C12Q-0001	**22.96**	*0*.*05*	0.80
	G01N-0033	**22.53**	*0*.*13*	0.43
	C12N-0015	**21.27**	*-0*.*02*	0.94
	A61K-0039	**18.52**	-0.48	0.02
	A61K-0038	**15.99**	-0.39	0.25
	A61K-0031	**13.92**	*0*.*67*	0.14
	C07K-0016	**12.36**	-0.28	0.04
	C12N-0005	**11.76**	*0*.*08*	0.73
	C07K-0014	**11.15**	-1.14	0.00
	A61K-0047	4.84	**0.48**	**0.00**
	A61K-0009	3.38	**0.47**	**0.00**
Electrical medical equipment	A61N-0005	**18.33**	**1.02**	**0.00**
(S05)	A61B-0005	**14.45**	*-0*.*15*	0.19
	A61B-0006	**11.15**	*0*.*46*	0.18
	A61B-0018	**9.46**	*-0*.*05*	0.85
Diagnosis, surgery	A61B-0005	**26.90**	*-0*.*49*	0.24
(P31)	A61B-0017	**20.98**	**1.17**	**0.00**
	A61B-0006	**18.11**	*0*.*52*	0.27
	A61B-0018	**17.10**	*-0*.*48*	0.24
